# Frequently Transmission and Close Relationship Among Immigrants in the China–Myanmar Border Region Indicated by Molecular Transmission Analysis From a Cross-Sectional Data

**DOI:** 10.3389/fmed.2021.693915

**Published:** 2022-04-27

**Authors:** Zhili Hu, Yingjie Liu, Jibao Wang, Zhefeng Meng, Sequoia I. Leuba, Jie Wei, Xing Duan, Zhenxing Chu, Min Chen, Hong Shang, Junjie Xu

**Affiliations:** ^1^NHC Key Laboratory of AIDS Immunology (China Medical University), National Clinical Research Center for Laboratory Medicine, The First Affiliated Hospital of China Medical University, Shenyang, China; ^2^Key Laboratory of AIDS Immunology, Chinese Academy of Medical Sciences, Shenyang, China; ^3^Key Laboratory of AIDS Immunology of Liaoning Province, Shenyang, China; ^4^Collaborative Innovation Center for Diagnosis and Treatment of Infectious Diseases, Hangzhou, China; ^5^Department of STD/AIDS Prevention and Control, Dehong Prefecture Center for Disease Control and Prevention, Mangshi, China; ^6^Key Laboratory of Digestive Cancer Full Cycle Monitoring and Precise Intervention of Shanghai Municipal Health Commission, Minhang Hospital, Fudan University, Shanghai, China; ^7^Department of Epidemiology, University of North Carolina at Chapel Hill, Chapel Hill, NC, United States; ^8^Institute for AIDS/STD Control and Prevention, Yunnan Center for Disease Control and Prevention, Kunming, China

**Keywords:** HIV, molecular epidemiology, phylogenetic analysis, cross-border, subtype

## Abstract

**Background:**

Accurate identification of molecular transmission clusters (MTCs) and understanding the dynamics of human immunodeficiency virus (HIV) transmission are necessary to develop targeted interventions to prevent HIV transmission. We evaluated the characteristics of antiretroviral therapy-naïve individuals who belonged to HIV-1 MTCs in the China–Myanmar border region to inform targeted effective HIV intervention.

**Methods:**

Phylogenetic analyses were undertaken on HIV-1 *pol* sequences to characterize subtypes or circulating recombinant forms and identify MTCs. MTCs were defined as those with 2 or more sequences having bootstrap support > 80% and a pairwise gene distance less than or equal to 0.03. Factors correlated with MTCs were evaluated using logistic regression analysis. The chi-square test was used to compare differences between Chinese and Burmese participants belonging to MTCs.

**Results:**

A total of 900 people had their *pol* gene successfully sequenced. Twenty-one MTCs were identified and included 110 individuals (12.2%). Individuals in MTCs were more likely to be Burmese [aOR = 2.24 (95% CI: 1.33, 3.79), *P* = 0.003], be younger [aOR = 0.34 (95% CI: 0.20, 0.58), *P* < 0.001 for age 26–50 vs. 25 years or younger], have a lower CD4 T cell count [aOR = 2.86 (95% CI: 1.34, 6.11), *P* = 0.007 for < 200 vs. 350 or greater], and have subtypes CRF07_BC or C [CRF07_BC: aOR = 7.88 (95% CI: 3.55, 17.52), *P* < 0.001; C: aOR = 2.38 (95% CI: 1.23, 4.62), *P* = 0.010 compared to CRF01_AE]. In MTCs, Burmese were younger (89.7 vs. 57.7% for age 25 years or younger), had a lower education level (41.0 vs. 8.5% for illiterate), were more likely to be infected through injection drug use (35.9 vs. 12.7%), and had a higher proportion of subtype BC (33.3 vs. 15.5%) and CRF01_AE (20.5 vs. 8.5%) compared to Chinese (*P* < 0.05 for all).

**Conclusion:**

Burmese participants were more likely to belong to MTCs, and most MTCs had both Burmese and Chinese participants. These data highlight the bidirectional transmission of HIV-1 frequently transmission and close relationship among immigrants in the China–Myanmar border region. Local health departments should pay more attention to HIV screening and intervention to immigrants Burmese with the characteristics of younger age, having lower CD4 T cell count and infected with HIV subtypes CRF07_ BC or C.

## Introduction

Human immunodeficiency virus (HIV) infection is a major global public health disease and can be spread through population mobility (1). Researchers can trace these migrating HIV strains to specific geographical regions and modes of transmission (2, 3). In border areas, the population mobility of transnational floating populations, including high-risk HIV key populations (e.g., injection drug users and sex workers), can drive HIV transmission between countries (4). Thus, multiple studies have focused on preventing HIV transmission in these border regions. A study on the US-Mexico border region has shown that there was bi-directional and cross-border HIV transmission (5). In addition, clustering with neighboring countries was found between 10.1% of people newly diagnosed with HIV-1 in Europe (6). As China shares borders with multiple countries, and previous research has shown that the HIV prevalence rate among transnational floating populations at Chinese frontier ports is significantly higher than the general population, prevention of HIV transmission across borders in China is critical (4).

Yunnan Province is the one of the most affected provinces by HIV/AIDS in China (7). More than 93,000 people were living with HIV/AIDS (PLWHA) in Yunnan in 2016, accounting for 14.3% of the total number in China (8). Located in southern China, Yunnan Province borders Myanmar in the west and Laos and Vietnam in the south and is near the “Golden Triangle,” a large narcotic production area. Due to its location and high HIV prevalence, Yunnan serves as a gateway for HIV transmission between Southeast Asia and China (9–11). In recent years, China and Myanmar have focused on developing strong trade and personnel exchanges, and many Burmese live, work, and study in Yunnan (12); almost 16 million Burmese annually enter Yunnan (13). In 2010, the Chinese State Council ended mandatory HIV/AIDS testing of returning Chinese and foreign visitors (14). Thus, the HIV infection status of this transnational population in Yunnan is mainly unknown (14). While incident cases of HIV/AIDS in Yunnan are decreasing, the proportion of newly diagnosed with HIV/AIDS in Yunnan province who are Burmese is almost 60% and increasing annually in Yunnan (4). Therefore, it is essential to identify and address migrating HIV strains spread among transnational floating populations to address the HIV epidemic in Yunnan.

One way to describe and monitor local HIV strains is through phylogenetic analysis, i.e., determining associations between genetic sequences and information on nationality, infection route, and drug resistance to determine evolutionary changes over time (15, 16). Phylogenetic cluster analysis can identify potential factors promoting the epidemic spread and likely transmission networks (17–19). Researchers in Beijing (20) have previously shown that targeting individuals for antiretroviral therapy (ART) using phylogenetic cluster analysis had a much higher prevention efficiency of future infections (42%) compared to providing all participants ART (24%). However, most studies conducted on phylogenetic analysis in Yunnan focus on subtype distribution and identifying drug resistance or new subtypes instead of assessing the associations between demographic and clinical factors and molecular transmission clusters (MTCs) (21–24).

We conducted a molecular phylogenetic analysis of an HIV-1 sequence dataset from the China-Myanmar border region to identify and characterize MTCs in the Yunnan. We also compared the differences between Chinese and Burmese clusters of ART-naive individuals to help target HIV strategies to prevent further transmission.

## Materials and Methods

### Study Population

**FIGURE 1:**
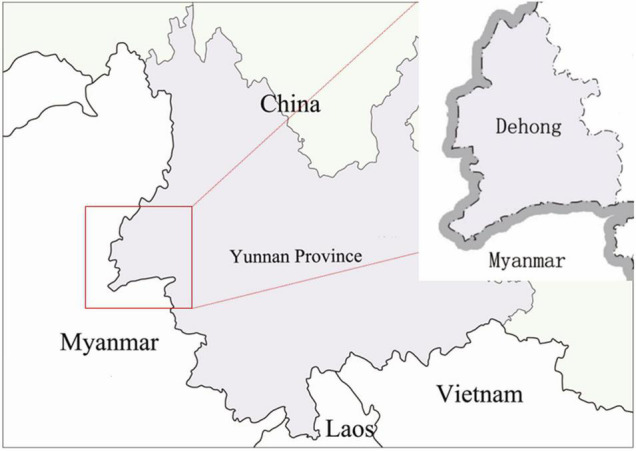
Study site location at the China-Myanmar border. The black dotted line with a gray shadow shows the border between China and Myanmar, and the light purple area indicates the region of Yunnan Province. The red box indicates our study site. Magnified images of boxed areas are shown in the upper right corners.

In Dehong Prefecture in Yunnan Province, 900 individuals newly reported with HIV had their *pol* sequences successfully amplified from 2009 to 2017 (Figure 1). Participants were recruited by advertising in local HIV/AIDS clinics and peer referral. Participants were eligible for this study if they were (1) infected with HIV-1 by western blot confirmatory test, and (2) had not started antiretroviral therapy (ART) and were consent to attend this study. Participants were asked about their epidemiological history through face-to-face interviews, and were collected whole blood samples (10 mL).

### HIV-1 RNA Extraction and *pol* Gene Amplification

HIV-1 RNA was extracted from plasma using the QIAamp Viral RNA Mini kit (Qiagen, Valencia, CA, United States) according to the manufacturer’s instructions. The One-Step RNA polymerase chain reaction (PCR) Kit (TaKaRa Biotechnology, Shiga, Japan) was used for reverse transcription and the first round of amplification of *pol*. The nested PCR was performed using 2 × Taq PCR Master Mix (Tiangen, Beijing, China). The primers, conditions and procedures for reverse transcription PCR were described in a previous study (23). The PCR product was identified by electrophoresis using 1% agarose gels and amplified PCR products were sent to Sino Geno Max (Beijing, China) for sequencing.

### HIV-1 Genotyping and Molecular Transmission Cluster Identification

Sequences were assembled using Sequencer 5.1 (Gene Codes, Ann Arbor, MI, United States). The assembled sequences were aligned with BioEdit 7.0.5.3 and then edited manually. Reference sequences for HIV-1 subtyping were selected and downloaded from the HIV databases of the Los Alamos National Laboratory.^1^ Neighbor-joining phylogenetic trees were constructed using MEGA 7.0,^2^ with the Kimura 2-parameter model with 1,000 bootstrap replicates to determining the HIV-1 genotype. The possible inter-subtype recombination was analyzed with the Recombination Identification Program (RIP).^3^ Molecular transmission clusters (MTCs) were defined as clusters with 2 or more sequences having bootstrap support greater than 80% (the criterion for phylogenetic confidence) and a pairwise gene distance less than or equal to 0.03 (the pairwise gene distance was calculated using the Tamura–Nei parameter model and setting the bootstrap to 1,000 with MEGA 7.0) (25–27).

### Statistical Analysis

Demographic and clinical data are summarized using median and interquartile ranges (IQR) for continuous variables or absolute and relative frequencies for categorical variables. Differences in characteristics of Chinese or Burmese who were included in MTCs were compared using the Chi-square test. Univariable and multivariable logistic regression models were used to determine correlations between potential demographic and clinical factors (e.g., sex, age, ethnicity, nationality, occupation, marital status, infection routes, CD4 cell count, and subtype) and inclusion in a molecular transmission cluster (MTC). The multivariable analysis was conducted using forward stepwise regression (entry criteria: *P* < 0.05; exit criteria: *P* > 0.1) to select the variables. Continuous variables such as age and CD4 cell count were categorized for statistical analysis. All statistical analyses were conducted using SPSS 25.0 (IBM, Armonk, NY, United States). *P* < 0.05 (two-sided) was considered significant.

### Ethical Approval of the Study Protocol

The Institutional Review Board of the First Affiliated Hospital of China Medical University (Shenyang, China) approved the study protocol (2019-152-3). Adults provided written informed consent before collection of blood samples; guardians signed the consent forms of adolescents.

## Results

### Characteristics of Participants

The study enrolled 900 individuals who had an HIV-1 *pol* region successfully sequenced. Most participants were male (59.9%, 539/900) and non-Han ethnicity (56.2%, 506/900), about 16.6% (149/900) were Burmese living in Yunnan. The median age was 28 years (IQR: 23, 39). Less than half had graduated from junior middle school or above (42.9%, 386/900), most identified their occupation as peasant (70.6%, 635/900), and about half were married (51.6%, 464/900). The median CD4 cell count was 440 (IQR: 324, 617). Most participants were infected with HIV through heterosexual contact (76.1%, 685/900), followed by injection drug use (IDU) (17.3%, 156/900) and homosexual contact (4.7%, 42/900) (Table 1).

**TABLE 1:** Demographic and clinical characteristics on participants, stratified by nationality (*N* = 900).

Characteristics	Total (*n* = 900) n (%)	China (*n* = 751) n (%)	Myanmar (*n* = 149) n (%)
**Age (years)**			
Median (IQR)	28 (23, 39)	30 (24, 42)	22 (20, 24)
≤ 25	384 (42.7)	249 (33.2)	135 (90.6)
26–50	428 (47.6)	417 (55.5)	11 (7.4)
> 50	88 (9.8)	85 (11.3)	3 (2.0)
**Sex**			
Male	539 (59.9)	449 (59.8)	90 (60.4)
Female	361 (40.1)	302 (40.2)	59 (39.6)
**Ethnicity**			
Han	394 (43.8)	346 (46.1)	48 (32.2)
Non-Han	506 (56.2)	405 (53.9)	101 (67.8)
**Education**			
Illiterate	170 (18.9)	104 (13.8)	66 (44.3)
Primary school	344 (38.2)	295 (39.3)	49 (32.9)
Junior middle school	280 (31.1)	253 (33.7)	27 (18.1)
High school and above	106 (11.8)	99 (13.2)	7 (4.7)
**Occupation**			
Peasant	635 (70.6)	531 (70.7)	104 (69.8)
Other	265 (29.4)	220 (29.3)	45 (30.2)
**Marital status**			
Single*	436 (48.4)	352 (46.9)	84 (56.4)
Married	464 (51.6)	399 (53.1)	65 (43.6)
**Infection route**			
Heterosexual	685 (76.1)	585 (77.9)	100 (67.1)
Homosexual	42 (4.7)	38 (5.1)	4 (2.7)
Injection drug use	156 (17.3)	113 (15.0)	43 (28.9)
Other^†^	17 (1.9)	15 (2.0)	2 (1.3)
**CD4 cell count (cell/μ L)**			
Median (IQR)	440 (324, 617)	439 (330, 617)	451 (316, 617)
<200	57 (6.3)	47 (6.3)	10 (6.7)
200–349	212 (23.6)	173 (23.0)	39 (26.2)
≥350	631 (70.1)	531 (70.7)	100 (67.1)
**HIV subtype**			
CRF01_AE	166 (18.4)	136 (18.1)	30 (20.1)
CRF07_BC	72 (8.0)	68 (9.1)	4 (2.7)
CRF08_BC	180 (20.0)	175 (23.3)	5 (3.4)
BCı	176 (19.6)	118 (15.7)	58 (38.9)
C	257 (28.6)	210 (28.0)	47 (31.5)
B	49 (5.4)	44 (5.9)	5 (3.4)

*Includes unmarried, divorced and widowed.

*^†^Includes mother to child transmission and unknown.*

### Phylogenetic Analysis

**FIGURE 2:**
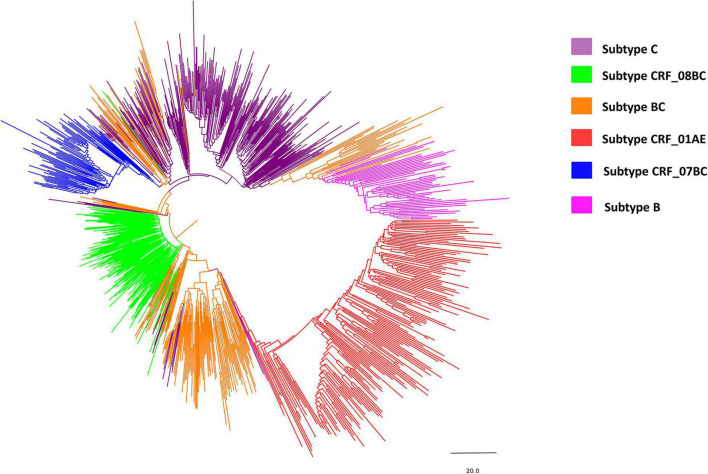
Neighbor-joining phylogenetic tree of the whole pol gene. The neighbor-joining phylogenetic tree was constructed using MEGA 7.0 with 1,000 bootstrap replicates. The nucleotide substitution model was the Kimura 2-parameter model. The line with different colors indicates different subtypes/recombinant forms of HIV-1. Purple, green, orange, red, blue and magenta lines denote subtype C, CRF_08BC, BC, CRF_01AE, CRF_07BC, and subtype B, respectively.

Phylogenetic analysis of blood samples found that individuals (28.6%; 257/900) were frequently infected with subtype C, followed by CRF08_BC (20.0%; 180/900), BC (19.6%; 176/900), CRF01_AE (18.4%; 166/900), CRF07_BC (8.0%; 72/900), and B (5.4%; 49/900) (Figure 2 and Table 1), and that the distribution of subtypes differed by country. Chinese participants were frequently infected with HIV subtype C (28.0%; 210/751), followed by subtype CRF08_BC (23.3%; 175/751), CRF01_AE (18.1%; 136/751) and BC (15.7%; 118/751). In contrast, Burmese participants were frequently infected with HIV subtype BC (38.9%; 58/149), followed by subtype C (31.5%; 47/149) and CRF01_AE (20.1%; 30/149).

### Molecular Transmission Clusters

**FIGURE 3:**
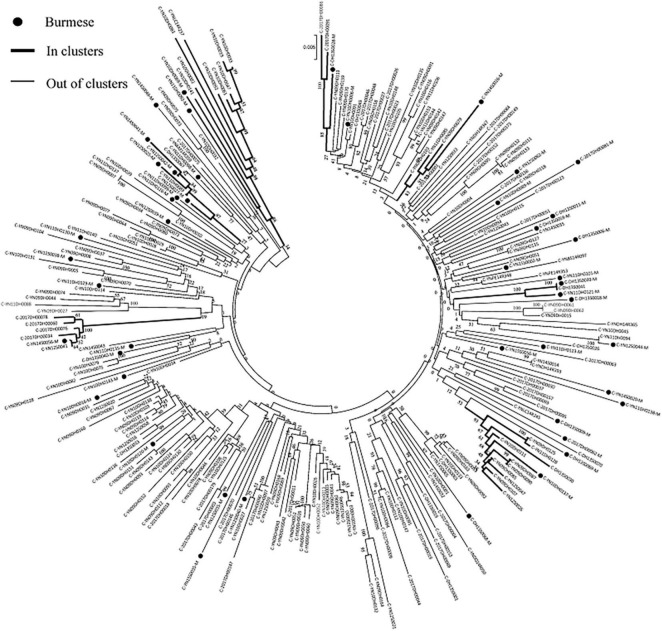
Clustering of HIV-1 sequences within the biggest molecular transmission clusters for subtypes C. The tree was constructed using MEGA 7.0 with 1,000 bootstrap replicates. Molecular transmission clusters (MTC) were defined as clusters with two or more sequences and the pairwise gene distance less than or equal to 0.03 with bootstrap values ≥ 80%. The scale bar indicates 0.5% nucleotide sequence divergence. The black nodes represent Burmese. The thick line indicates individuals in clusters. The thin line indicates individuals out of clusters.

**FIGURE 4:**
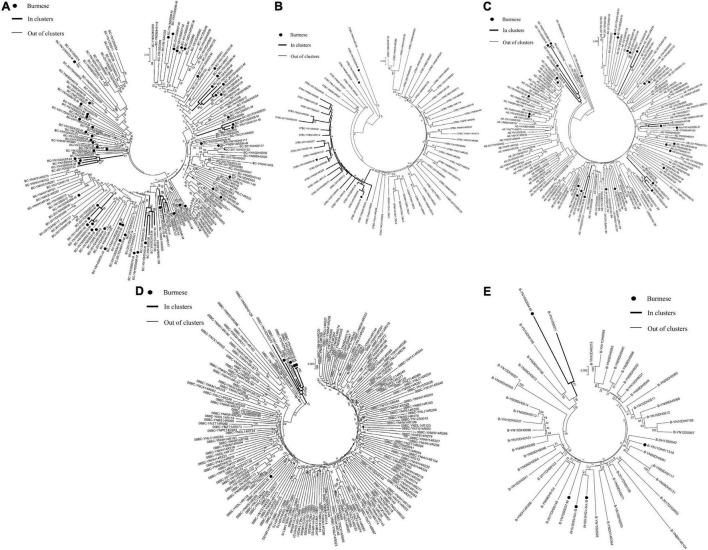
Clustering of HIV-1 sequences for other subtypes. The tree was constructed using MEGA 7.0 with 1,000 bootstrap replicates. Molecular transmission clusters (MTC) were defined as clusters with two or more sequences and the pairwise gene distance less than or equal to 0.03 with bootstrap values ≥ 80%. The scale bar indicates 0.5% nucleotide sequence divergence. The black nodes represent Burmese. The thick line indicates individuals in clusters. The thin line indicates individuals out of clusters. **(A)**, subtype BC; **(B)**, subtype CRF_07BC; **(C)**, subtype CRF_01AE; **(D)**, subtype CRF_08BC; **(E)**, subtype B.

Phylogenetic analyses identified 21 MTCs involving 12.2% (110/900) of participants. The clustering rate among Chinese participants was 9.5% (71/751), and among Burmese participants was 26.2% (39/149). Most MTCs (95.2%, 20/21) consisted of sequences from China and Myanmar, whereas only one MTC (4.8%) consisted exclusively of sequences from Myanmar. The highest proportion of MTC sequences belonged to subtypes C (42/110; 38.2%), BC (24/110; 21.8%), and CRF07_BC (22/110; 20.0%) (Table 2 and Figures 3, 4).

**TABLE 2:** Demographic and clinical characteristics of individuals included in the MTCs stratified by nationality (*N* = 110).

Characteristics	Overall (*N* = 110) n (%)	Chinese (*n* = 71) n (%)	Burmese (*n* = 39) n (%)	*P*-value^a^
**Age (years)**				
≤25	76 (69.1)	41 (57.7)	35 (89.7)	0.002
26–50	28 (25.5)	24 (33.8)	4 (10.3)	
>50	6 (5.5)	6 (8.5)	0 (0.0)	
**Sex**				
Male	63 (57.3)	39 (54.9)	24 (61.5)	0.503
Female	47 (42.7)	32 (45.1)	15 (38.5)	
**Ethnicity**				
Han	50 (45.5)	35 (49.3)	15 (38.5)	0.275
Non-Han	60 (54.5)	36 (50.7)	24 (61.5)	
**Education**				
Illiterate	22 (20.0)	6 (8.5)	16 (41.0)	<0.001
Primary school	30 (27.3)	21 (29.6)	9 (23.1)	
Junior middle school	44 (40.0)	35 (49.3)	9 (23.1)	
High school and above	14 (12.7)	9 (12.7)	5 (12.8)	
**Occupation**				
Peasant	71 (64.5)	44 (62.0)	27 (69.2)	0.446
Other	39 (35.5)	27 (38.0)	12 (30.8)	
**Marital status**				
Single*	60 (54.5)	36 (50.7)	24 (61.5)	0.275
Married	50 (45.5)	35 (49.3)	15 (38.5)	
**Infection route**				
Heterosexual	74 (67.3)	51 (71.8)	23 (59.0)	0.021
Homosexual	11 (10.0)	9 (12.7)	2 (5.1)	
Injection drug use	23 (20.9)	9 (12.7)	14 (35.9)	
Other^†^	2 (1.8)	2 (2.8)	0 (0.0)	
**CD4 cell count (cells/μ L)**				
<200	12 (10.9)	7 (9.9)	5 (12.8)	0.634
200–349	30 (27.3)	18 (25.4)	12 (30.8)	
≥350	68 (61.8)	46 (64.8)	22 (56.4)	
**HIV subtype**				
CRF01_AE	14 (12.7)	6 (8.5)	8 (20.5)	0.001
CRF07_BC	22 (20.0)	20 (28.2)	2 (5.1)	
CRF08_BC	6 (5.5)	2 (2.8)	4 (10.3)	
BCı	24 (21.8)	11 (15.5)	13 (33.3)	
C	42 (38.2)	31 (43.7)	11 (28.2)	
B	2 (1.8)	1 (1.4)	1 (2.6)	

*Includes unmarried, divorced and widowed.

*^†^Includes mother to child transmission and unknown.*

*^a^Chi-squared test.*

### Comparison of Characteristics of Individuals in Molecular Transmission Clusters

Individuals included in the MTCs were stratified based on nationality: 71 Chinese and 39 Burmese. Burmese were more likely to be younger (25 years or younger: 89.7 vs. 57.7%), have a lower levels of education (illiterate: 41.0 vs. 8.5%), have been infected through IDU (35.9 vs. 12.7%), and have HIV subtype BC (33.3 vs. 15.5%) or subtype CRF01_AE (20.5 vs. 8.5%) compared to Chinese participants (*P* < 0.05 for all) (Table 2).

### Factors Associated With Being Grouped With an Molecular Transmission Clusters

Based on the results from the univariable logistic regression, older participants were less likely to be in MTCs [age 26–50 years: OR = 0.28 (95% CI: 0.18, 0.45); age > 50 years: OR = 0.30 [95% CI: 0.12, 0.71] compared to age 25 years or younger]. Participants with HIV subtype CRF07_BC [OR = 4.78 (95% CI: 2.27, 10.04)], CRF08_BC [OR = 0.37 (95% CI: 0.14, 1.00)] or HIV subtype C [OR = 2.12 (95% CI: 1.12, 4.02)] were more likely to be grouped into an MTC when compared to individuals with subtype CRF01_AE. Being Burmese compared to being Chinese [OR = 3.40 (95% CI: 2.19, 5.27)], being infected through homosexual sex compared to through heterosexual sex [OR = 2.93 (95% CI: 1.41, 6.07)], and having a CD4 cell count below 200 compared to 350 or greater [OR = 2.21 (95% CI: 1.13, 4.38)] were associated with increased likelihood of being grouped into an MTC (Table 3).

**TABLE 3:** Univariable and multivariable logistic regression of factors associated with being grouped with a molecular transmission cluster (MTC).

Characteristics	Non-clustered (*n* = 790) n (%)	Clustered (*n* = 110) n (%)	Univariable analysis		Multivariable analysis	
			OR (95% CI)	*P*-value	aOR (95% CI)	*P*-value
**Age (years)**						
≤ 25	308 (39.0)	76 (69.1)	Ref.		Ref.	
26–50	400 (50.6)	28 (25.5)	0.28 (0.18, 0.45)	<0.001	0.34 (0.20, 0.58)	<0.001
> 50	82 (10.4)	6 (5.5)	0.30 (0.12, 0.71)	0.006	0.40 (0.16, 1.01)	0.052
**Sex**						
Male	476 (60.3)	63 (57.3)	Ref.			
Female	314 (39.7)	47 (42.7)	1.13 (0.76, 1.69)	0.550		
**Ethnicity**						
Han	344 (43.5)	50 (45.4)	Ref.			
Non-Han	446 (56.5)	60 (54.5)	0.93 (0.62, 1.38)	0.705		
**Nationality**						
China	680 (86.1)	71 (64.5)	Ref.		Ref.	
Myanmar	110 (13.9)	39 (35.5)	3.40 (2.19, 5.27)	<0.001	2.24 (1.33, 3.79)	0.003
**Education**						
Illiterate	148 (18.7)	22 (20.0)	Ref.			
Primary school	314 (39.7)	30 (27.3)	0.64 (0.36, 1.15)	0.138		
Junior middle school	236 (29.9)	44 (40.0)	1.25 (0.72, 2.18)	0.421		
High school and above	92 (11.6)	14 (12.7)	1.02 (0.50, 2.10)	0.949		
**Occupation**						
Peasant	564 (71.4)	71 (64.5)	Ref.			
Other	226 (28.6)	39 (35.5)	1.37 (0.90, 2.09)	0.141		
**Marital status**						
Single*	376 (47.6)	60 (54.5)	Ref.			
Married	414 (52.4)	50 (45.5)	0.76 (0.51, 1.13)	0.173		
**Infection route**						
Heterosexual	611 (77.3)	74 (67.3)	Ref.			
Homosexual	31 (3.9)	11 (10.0)	2.93 (1.41, 6.07)	0.004		
Injection drug use	133 (16.8)	23 (20.9)	1.43 (0.86, 2.36)	0.166		
Other^†^	15 (1.9)	2 (1.8)	1.10 (0.25, 4.91)	0.900		
**CD4 cell count (cells/μ L)**						
< 200	45 (5.7)	12 (10.9)	2.21 (1.13, 4.38)	0.023	2.86 (1.34, 6.11)	0.007
200–349	182 (23.0)	30 (27.3)	1.36 (0.86, 2.16)	0.186	1.46 (0.89, 2.39)	0.137
≥ 350	563 (71.3)	68 (61.8)	Ref.		Ref.	
**HIV subtype**						
CRF01_AE	152 (19.4)	14 (12.7)	Ref.		Ref.	
CRF07_BC	50 (6.3)	22 (20.0)	4.78 (2.27, 10.04)	<0.001	7.88 (3.55, 17.52)	<0.001
CRF08_BC	174 (22.0)	6 (5.5)	0.37 (0.14, 1.00)	0.050	0.68 (0.25, 1.88)	0.460
BCı	152 (19.2)	24 (21.8)	1.71 (0.85, 3.44)	0.129	1.49 (0.72, 3.08)	0.283
C	215 (27.2)	42 (38.2)	2.12 (1.12, 4.02)	0.021	2.38 (1.23, 4.62)	0.010
B	47 (5.9)	2 (1.8)	0.46 (0.10, 2.11)	0.319	0.63 (0.13, 2.96)	0.559

*Includes unmarried, divorced and widowed.

*^†^Includes mother to child transmission and unknown.*

*aOR: adjusted sex, ethnicity, education, occupation, and marital status.*

In the multivariable logistic regression using forward stepwise regression, we adjusted for sex, occupation, education, and marital status variables. Participants were more likely to be grouped into an MTC if they were Burmese compared to Chinese [aOR = 2.24 (95% CI: 1.33, 3.79)], have a CD4 cell count below 200 compared to 350 or greater [aOR = 2.86 (95% CI: 1.34, 6.11)], or have HIV subtype CRF07_BC [aOR = 7.88 (95% CI: 3.55, 17.52)] or subtype C [aOR = 2.38 (95% CI: 1.23, 4.62)] compared to subtype CRF01_AE. Participants were less likely to be grouped into an MTC if they were between 26 and 50 years old compared to 25 years old or younger [aOR = 0.34 (95% CI: 0.20, 0.58)] (Table 3).

## Discussion

We evaluated 900 ART-naïve individuals to determine the characteristics and distribution of HIV-1 molecular transmission clusters (MTCs) in the China–Myanmar border region using phylogenetic analyses. We found that 12.2% of the HIV-1 sequences belonged in MTCs, and that the factors associated with belonging to MTCs were younger age, Burmese nationality, lower CD4 cell count, and HIV-1 subtype of CRF07_BC or subtype C compared to subtype CRF01_AE. Among those included in MTCs, Burmese were younger, had a lower education level, and more were infected through IDU compared to Chinese. These results provide evidence of the molecular epidemiology of HIV-1 in the southwest border region needed for public-health policy and targeted interventions to prevent HIV-1 transmission in the China–Myanmar border region.

We found that 12.2% of HIV-1 *pol* sequences included in this study belonged in MTCs, which is lower than that reported locally (33% in 2015–2016) (28). We may have found a lower percentage belonging to MTCs by having an earlier and longer study period (2009–2017 vs. 2015–2016). Similar to previous published results, we found that younger age was correlated with belonging to MTCs, suggesting that local government should target HIV prevention strategies toward younger people living near the China–Myanmar border (29, 30). While only 6.3% (57/900) of participants had a CD4 cell count below 200, 21.1% (12/57) of these participants with low CD4 cell counts belonged to MTCs [aOR = 2.86 (95% CI: 1.34, 6.11)]. In contrast, a previous study found that high, not low, CD4 cell count was more likely to belong to MTCs (30). Our conflicting result may be explained that due to low HIV testing, many people who are diagnosed with HIV in Yunnan may be diagnosed at a later stage and have lower CD4 cell counts at diagnosis (31). Thus, local HIV testing must be expanded to reach these delayed diagnosed individuals to reduce transmission of HIV.

We also found that specific HIV subtypes were correlated with belonging to MTCs. Subtype CRF07_BC and subtype C were more likely to belong to MTCs than CRF01_AE (20.0 and 38.2 vs. 12.7%), suggesting that the two subtypes were more likely to have genetically close infections in Yunnan. Subtype C was the most prevalent subtype in this dataset (28.6%), followed by subtypes CRF08_BC (20.0%), BC (19.6%), and CRF01_AE (18.4%), consistent with previous reports of the main local HIV subtypes (2, 32–35). Similar to previous studies, we also found differences in primary subtype among Chinese and Burmese (21, 36, 37). Subtypes C, CRF08_BC, and CRF01_AE were the most prevalent HIV subtypes present among Chinese, while subtypes BC, C, and CRF01_AE were the most prevalent among Burmese. These different distributions of subtype based on nationality may be correlated with the high prevalence of HIV-1 recombinants among northern Burmese (38–40). We found more CRFs among Chinese not Burmese and the high prevalence of CRFs among Burmese. This result might suggest the frequently transmission of HIV-1 in the China–Myanmar border region.

While Burmese comprised only 16.6% of the study cohort, 35.5% (39/110) were in MTCs and Burmese had more than twice the odds of belonging to MTCs compared to Chinese, suggesting that Burmese participants from Yunnan had higher odds of transmitting HIV. Most MTCs included both Burmese and Chinese participants, and only one MTC was comprised only of Burmese, suggesting cross-border HIV transmission. Among participants belonging to MTCs, Burmese participants were younger (25 years old or younger: 89.7 vs. 57.7%), had more illiterate education (41.0 vs. 8.5%), and more HIV infections through IDU (35.9 vs. 12.7%) and fewer HIV infections through homosexual behavior (5.1 vs. 12.7%) compared to Chinese participants. These demographic differences among participants belonging to MTCs based on nationality were also found among all participants. These findings suggest that local governments should take different targeted approaches depending on nationality to prevent and control HIV transmission in the China–Myanmar region such as providing treatment and support for reducing injection drug use among Burmese and increasing condom use or pre-exposure prophylaxis among Chinese in Yunnan in order to address the main infection routes for HIV acquisition.

To the best of our knowledge, we are the first to determining clinical and demographic factors correlated with MTCs between antiretroviral therapy-naïve Chinese and Burmese in the China–Myanmar border region. We found that younger age, Burmese nationality, lower CD4 cell count, and having subtype CRF07_BC or subtype C (compared to subtype CRF01_AE) were significantly correlated with belonging to MTCs. Our findings support local CDC conducting targeted interventions addressing cross-border HIV transmission in the China-Myanmar border, in particular to local young Burmese. Lower CD4 cell count was significantly correlated with belonging to MTCs, which indicates that some delayed diagnosed PLWHA is still transmitting HIV, hence continue expanding local HIV testing is also necessary to curb the local HIV transmission. Meanwhile, we identified 21 MTCs totally, but only one MTC was comprised only of Burmese and we found popular HIV subtypes were similar between Chinese and Burmese in the China–Myanmar border region. This result might suggest the frequently transmission and close relationship among immigrants. Hence we need to further strengthen the surveillance of HIV clustering characteristics in China-Myanmar border region.

However, although seven other prefectures in Yunnan bordered other countries, participants were only recruited from one prefecture: Dehong prefecture. However, Dehong prefecture has the highest prevalence of HIV-1 infections compared to the other prefectures and thus may suggest a pattern found in other prefectures in Yunnan (41). Second, we could not infer the direction of transmission through phylogenetic analyses and the cross-sectional study design has limited causal inferences and the representativeness and generalizability of our study are uncertain owing to the small number of samples. Finally, despite the extensive use of pol as a main target to reconstruct transmission clusters, the more genes and the longer the alignment, the better the phylogenetic signal and the better the tree resolution. Despite these limitations, we characterized HIV-1 transmission in the China-Myanmar border region and provided evidence for targeted interventions to address cross-border transmission.

## Conclusion

We were able to identify 21 MTCs of which 20 included both Burmese and Chinese participants. Burmese were more likely to belong to MTCs compared to Chinese. Among those clustered, Burmese were more likely to be younger, have lower education, and be infected through injection drug use compared to Chinese participants. In contrast, among those who belonged to MTCs, Chinese participants were more likely to be infected through homosexual behavior. These data highlight the frequently transmission and close relationship among immigrants in the China–Myanmar border region, and suggest that the local government develop targeted interventions stratified by nationality to reduce cross-border HIV transmission.

## Data Availability Statement

The data analyzed in this study is subject to the following licenses/restrictions: data not publicly available but may be obtained upon request and approval from a third party, NHC Key Laboratory of AIDS Immunology (China Medical University). Requests to access these datasets should be directed to JX, xjjcmu@163.com.

## Ethics Statement

The studies involving human participants were reviewed and approved by the Institutional Review Board of the First Affiliated Hospital of China Medical University (2019-152-3). Written informed consent to participate in this study was provided by the participants or their legal guardian/next of kin.

## Author Contributions

ZH, YL, JBW, ZM, MC, HS, and JX conceived and designed the study. JBW, XD, and ZC performed the study. JBW and XD collected the data. ZH, YL, JW, and ZM analyzed the data. ZH, ZM, and YL drew the figures and tables. ZH wrote the first draft of the manuscript. ZH, YL, JBW, ZM, SL, HS, MC, and JX revised the manuscript. All authors revised the manuscript, and approved the final draft.

## Conflict of Interest

The authors declare that the research was conducted in the absence of any commercial or financial relationships that could be construed as a potential conflict of interest. The handling editor declared a shared affiliation with one of the author ZM at the time of the review.

## Publisher’s Note

All claims expressed in this article are solely those of the authors and do not necessarily represent those of their affiliated organizations, or those of the publisher, the editors and the reviewers. Any product that may be evaluated in this article, or claim that may be made by its manufacturer, is not guaranteed or endorsed by the publisher.

## Footnotes

1
www.hiv.lanl.gov

2
www.megasoftware.net

3
https://www.hiv.lanl.gov/content/sequence/RIP/RIP.html

## Abbreviations

MTC: molecular transmission clusters
aOR: adjust odd ratio
PLWHA: people living with HIV/AIDS.
